# Effect of passive shoulder exoskeleton support during working with arms over shoulder level

**DOI:** 10.1017/wtc.2023.21

**Published:** 2023-11-03

**Authors:** Annina Brunner, Rachel van Sluijs, Tobias Luder, Cherilyn Camichel, Melanie Kos, Dario Bee, Volker Bartenbach, Olivier Lambercy

**Affiliations:** 1 Department of Health Sciences and Technology, ETH, Zurich, Switzerland; 2 Research and Development, Auxivo AG, Schwerzenbach, Switzerland

**Keywords:** muscle activity, cardiac cost, usability, occupational exoskeletons, preventive technology, passive exoskeleton, soft exoskeleton, textile spring

## Abstract

Musculoskeletal disorders have the highest prevalence of work-related health problems. Due to the aging population, the prevalence of shoulder pain in workers in physically demanding occupations is increasing, thereby causing rising costs to society and underlining the need for preventive technologies. Wearable support structures are designed to reduce the physical work load during physically demanding tasks. Here, we evaluate the physiological benefit of the DeltaSuit, a novel passive shoulder exoskeleton, using an assessment framework that conforms to the approach proposed in the literature.

In this study, 32 healthy volunteers performed isometric, quasi-isometric, and dynamic tasks that represent typical overhead work to evaluate the DeltaSuit performance. Muscle activity of the arm, neck, shoulder, and back muscles, as well as cardiac cost, perceived exertion, and task-related discomfort during task execution with and without the exoskeleton were compared.

When working with the DeltaSuit, muscle activity was reduced up to 56% (*p <* 0.001) in the Trapezius Descendens and up to 64% (*p <* 0.001) in the Deltoideus_medius_. Furthermore, we observed no additional loading on the abdomen and back muscles. The use of the exoskeleton resulted in statistically significant reductions in cardiac cost (15%, *p <* 0.05), perceived exertion (21.5%, *p <* 0.001), and task-related discomfort in the shoulder (57%, *p <* 0.001).

These results suggest that passive exoskeletons, such as the DeltaSuit, have the potential to meaningfully support users when performing tasks in overhead postures and offer a valuable solution to relieve the critical body parts of biomechanical strains for workers at high risk of musculoskeletal disorders.

## 1. Introduction

Musculoskeletal disorders have the highest prevalence of work-related health problems (Kok et al., 2019). These disorders can affect different body parts, depending on the occupations and involved tasks. While working with hands-on or above shoulder level (e.g., assembly during manufacturing, electric installation, or logistics), the biomechanical strains in the shoulder increase, compared to when the arms are below the shoulder level. The exertion of the muscle, tendon, and nerves in the shoulder, through prolonged overhead work, increases the risk of gradually developing musculoskeletal disorders (Barthelme et al., 2021). In Europe, 49% of the ISCO-08 occupation “craft and related trades workers” report musculoskeletal pains in the shoulder, neck, and/or upper limbs per year. With 48%, the largest age group is “55 and over” reporting shoulder pain (Kok et al., 2019). It has further been shown that the prevalence of shoulder pain continues to increase beyond the age of 50 in physically demanding occupations (Hodgetts et al., 2021), thereby causing tremendous costs to society (Virta et al., 2012) and underlining the need for solutions to prevent the onset of such musculoskeletal disorders (Barthelme et al., 2021).

One possibility to relieve the shoulder musculature of biomechanical strains is equipping workers at high risk of musculoskeletal disorders with support structures such as exoskeletons. These are wearable devices that support movement to reduce load on the musculoskeletal system. Multiple exoskeletons providing shoulder support are already on the market (De Bock et al., 2022). To keep the exoskeletons lightweight, comfortable, and low-cost, most existing systems are passive (Maurice et al., 2019). The support from these passive systems is provided by the deformation of springs or other elastic materials that can store energy and return it to the user (De Looze et al., 2016; van Sluijs et al., 2023). It is important to qualitatively and quantitatively investigate how these devices are affecting the user during work (De Vries and De Looze, 2019; De Bock et al., 2022).

Wearable support structures can be assessed in a validation, evaluation, or field study. Here we focus on an evaluation study, meaning investigating a device in a controlled setting with applied tasks (De Bock et al., 2022). In previous evaluations of passive shoulder support structures, isometric (Pacifico et al., 2020), quasi-isometric tasks such as power tool handling (Schmalz et al., 2019; Hyun et al., 2019) and dynamic working tasks such as lifting (Theurel et al., 2018; Pacifico et al., 2020; van der Have et al., 2022) were included. In most evaluations, objective measurements are included, such as muscle activity measured with surface electromyography and cardiac cost. Furthermore, subjective measurement with questionnaires is included to assess movement freedom, task and device-related discomfort, as well as device usability (De Bock et al., 2022). Previous studies have reported significant reductions in muscle activity, lower cardiac cost, and positive changes in subjective data while working with passive shoulder exoskeletons (Hyun et al., 2019; Pacifico et al., 2020; Schmalz et al., 2019; Theurel et al., 2018). However, until recently there was no consensus on assessment protocols in the young field of occupational exoskeletons, making the results reported in the literature challenging to compare. In recent years some benchmark recommendations have been created, where specific tasks, duration, and multi-domain outcome measures (objective and subjective) are suggested. These recommendations are important to follow so that evaluation protocols are consistent and performance indicators from different exoskeletons can be compared.

In this paper, we report on a study evaluating in detail a novel passive shoulder exoskeleton, the DeltaSuit, in isometric, quasi-isometric, and dynamic overhead work tasks while following benchmark recommendations for occupational overhead exoskeleton evaluation proposed by De Bock et al. (2022). Specifically, De Bock et al. (2022) reviewed the literature and analyzed the most commonly used protocols. Based on their findings, they give guidance on both the design of the task environment (standardized tasks, overhead arm position, weight of tools), as well as the measurement protocol (selection of muscles measured). Both objective measures, such as muscle activity and cardiac cost, and subjective measures were recorded to evaluate the physiological effect of wearing the passive shoulder exoskeleton. We hypothesize that working with the passive shoulder support provided by the exoskeleton leads to a reduction in shoulder muscle activity, reduces cardiac cost and lowers perceived exertion, as well as task-related discomfort. As a secondary objective, a correlation analysis between anthropometric measurements of the participants and their subjective feedback aims to identify potential adaptations to be made to the exoskeleton mechanism to optimize comfort and minimize constraint for users with a variety of body types and sizes.

## 2. Materials and methods

### 2.1. Participants

Volunteers of working age (18–65 years) were eligible for the study. Individuals who reported acute or historic joint or muscle pain or stiffness were excluded. Data from 32 healthy volunteers (15 women) aged between 20 and 65 years (mean: 26.7 years, SD: 10.2 years) were collected. In the sample, body height ranged from 160 cm to 194 cm (mean: 174 cm, SD: 8.8 cm), and body weight ranged between 43.8 kg and 98.1 kg (mean: 72.1 kg, SD: 13.5 kg).

### 2.2. Passive shoulder exoskeleton

**Figure 1.**
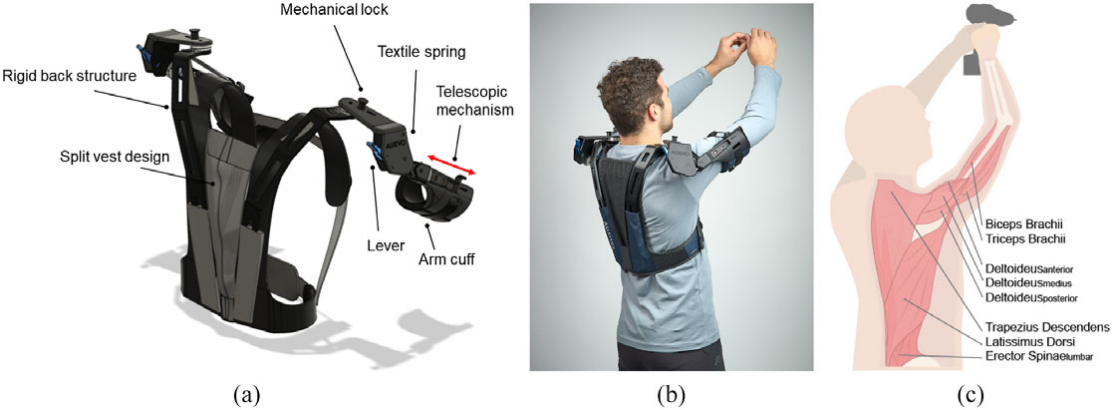
(a) Technical drawing of the DeltaSuit with the split vest design, the mechanical lock, the support mechanism, and the telescopic mechanism. (b) The DeltaSuit worn by a participant while doing overhead work. (c) Visual representation of the shoulder and back muscles that were measured with surface electromyography sensors. Additionally, the Rectus Abdominis muscle was also measured, although not marked in the picture.

In this study, a prototype of the DeltaSuit (Auxivo AG, Switzerland), a novel shoulder exoskeleton, was used (Figure 1(b)). The exoskeleton is worn like a vest and transmits force to its user through a textile interface connected to the user’s torso above the waist and through cuffs that attach to the arm above the elbow. The exoskeleton is designed to support the weight of the arms as well as an external load during work at or above shoulder level. The provided support can be adapted to the user’s need, with support level 1 (SL1) providing 5.2 Nm and support level 2 (SL2) providing 6.6 Nm peak flexion torque around the shoulder. This results in a peak supported weight of 4.2 kg depending on arm position and cuff placement. The design, which unlike most existing systems, does not go down to the user’s hips, is optimized for torso movement freedom. During donning and doffing, as well as during work where no shoulder support is needed, the arm cuffs can be stored behind the shoulder with a mechanical lock (Figure 1(a)).

The support mechanism of the proposed shoulder exoskeleton consists of textile springs located laterally at the level of each shoulder, supporting shoulder flexion. The textile spring is routed to provide a flexion-dependent support profile. Maximum support is provided when the arms are at 90° shoulder flexion, which corresponds to the maximal gravitational torque around the shoulder joint. Besides the supported degrees of freedom (DoF), the exoskeleton has a second passive DoF which rotates around a vertical axis, allowing unconstrained shoulder adduction/abduction. Shoulder elevation is allowed partially through the vest attachment to the upper torso only, and the omission of a connection between the shoulder structures and the hip. As these DoF of the exoskeleton do not perfectly replicate the DoF of the human shoulder joint, an additional telescopic joint with 6 cm range between the shoulder mechanism and the arm cuff compensates for misalignment. Therefore, we do not expect noticeable movement constraints during occupational tasks.

The exoskeleton is designed to fit users with t-shirt sizes S to XL and comes in two size options: S/M and L/XL. The textile vest, the rigid back structure, and the length of the upper arm structure vary with exoskeleton size. The vest is designed to auto-adjust to the users shoulder width through a vertical elastic fabric section arranged over the entire back length of the vest separating the left and right rigid structures (split vest design, Figure 1(a)). The exoskeleton further auto-adjusts to upper arm length through the telescopic mechanism. Additional size adaptability is provided through length adjustable bands in front of the chest. Due to the use of light textile springs and intensive use of textile components, the overall weight of the DeltaSuit could be reduced to 2.1 kg.

### 2.3. Study protocol

**Figure 2.**
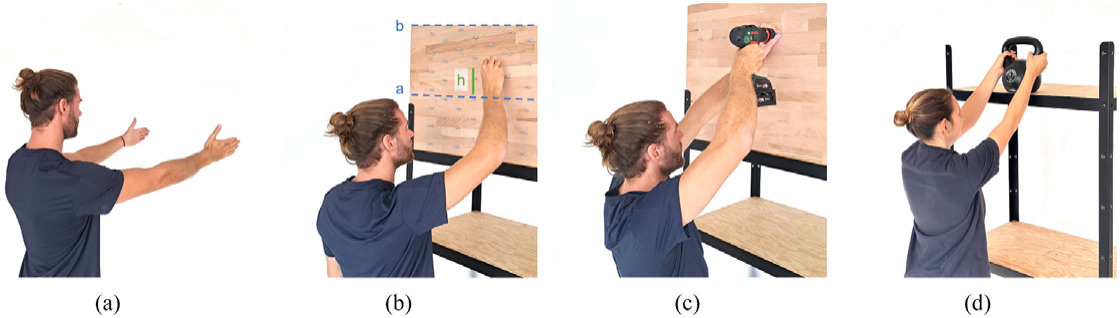
(a) Isometric task arm posture: 90° shoulder flexion with extended elbow, was held for 10 s. (b) Assembly task: participants assembled nuts without tool on overhead working height defined as h = *a* + 0.4(*b – a*) with [a] hand height with the shoulder and elbow flexed at 90° and [b] upper arm in full extension (Sood et al., 2007; De Bock et al., 2022) for 3 min. (c) Power tool handling task: participants used a power tool of 1.8 kg at the standardized individual overhead working height h for 3 min. (d) Load manipulation task: participants moved a load (♀: 8 kg / ♂: 12 kg) between two shelves at hip, respectively, overhead height h for 3 min.

Measurements took place during one visit to the Rehabilitation Engineering Laboratory of ETH Zurich. Upon arrival, the study protocol was explained and participants signed the informed consent sheet. Next, anthropometric measures, including torso length and shoulder width, were taken. After sensor placement (electromyography and heart rate, see Section 2.4), participants were fitted into the exoskeleton and the device was correctly adjusted with the help of the experimenter. Participants received a 10-min training on exoskeleton use, which included executing shortened versions of all study tasks. Next, normalization values for muscle activity (maximal voluntary contraction) and heart rate (resting heart rate) were obtained. The core experiment consisted of a series of standardized isometric, quasi-isometric and dynamic tasks, which were selected to represent a variety of relevant work scenarios (Figure 2). It is of interest to compare task performance without an exoskeleton (OFF) to task performance with the exoskeleton at SL1and SL2. For this purpose, the isometric task was performed with all three conditions in randomized order. However, to avoid development of fatigue during the protocol and to avoid a drop in participant attention due to prolonged duration of the protocol, the number of conditions for the quasi-isometric and dynamic tasks was limited to OFF and SL2. Overall, a typical measurement session lasted 90–120 min.

#### 2.3.1. Isometric task

The isometric task was designed to assess the effect of the two exoskeleton support levels on muscle activity in specific shoulder and overhead height arm postures. The task consists of five postures which were held for 10 s, with 10 s rest between postures: (i) 90° shoulder flexion with extended elbow (Figure 2(a)), (ii) 90° shoulder and elbow flexion, (iii) 120° shoulder flexion with an extended elbow, (iv) 90° shoulder and elbow flexion with a 45° horizontal abduction, (v) 120° shoulder flexion with 90° elbow flexion and 45° horizontal abduction. The posture of 90° shoulder flexion with an extended elbow joint is advised by De Bock et al., as it produces the maximal gravitational torque around the shoulder joint and is the most commonly used posture in literature (De Bock et al., 2022; Pacifico et al., 2020). The last two postures with a horizontal abduction angle were chosen to represent natural postures observed by the researchers during workplace assessments. To compare working without the exoskeleton to the two support levels provided by the DeltaSuit, the isometric task was performed with three exoskeleton conditions (OFF, SL1, and SL2) in randomized order.

#### 2.3.2. Assembly

During this quasi-isometric task, participants were instructed to assemble nuts onto bolts for a duration of 3 min (Figure 2(b)). The task resembles any occupation involving overhead assembly work without the use of a tool. The board with the bolts was individually adjusted to a proposed standardized overhead height, defined as h = *a* + 0.4(*b – a*) where [a] is the hand height with the shoulder and elbow flexed at 90° and [b] the hand height with the upper arm in full extension (Sood et al., 2007; De Bock et al., 2022). The task was performed in OFF and SL2 conditions in randomized order.

#### 2.3.3. Power tool handling

In this quasi-isometric task, participants were instructed to drive screws into a wood board with a power tool with their dominant hand for a duration of 3 min (Figure 2(c)). This represents work in multiple occupations, such as vehicle manufacturing. The power tool had a recommended tool weight of 1.8 kg (De Bock et al., 2022). The task was divided into two rounds, where each round consisted of 90 s of driving the screws into a wood board and 90 s of retrieving the screws from the board with the power tool. Also, for this task, the participants’ hands were on standardized individual overhead height (h). The task was performed in OFF and SL2 conditions in randomized order.

#### 2.3.4. Load manipulation

For this dynamic task, participants were instructed to lift and lower a load placed on a rack, from hip level to standardized individual overhead level h (Figure 2(d)). The task resembles work in logistics-related occupations. The box had a weight of 8 kg for female participants and 12 kg for male participants. In total, participants executed 18 cycles, each consisting of one lifting and lowering of the box. Participants were instructed to lift the box within a time frame of 5 s and lower it within the following 5 s. The individual lift and lowering movements were paced by a computer-generated voice from Delsys EMGworks software (Delsys Europe, United Kingdom). The task was performed in a randomized order in two conditions: OFF and SL2.

### 2.4. Data collection and processing

#### 2.4.1. Muscle activity

With the objective of evaluating the effect of the exoskeleton on agonist and antagonist muscles involved in overhead movements, while also ensuring that muscles normally not involved in the tasks are not affected, we recorded muscle activity from the upper arm, neck, shoulder, lower back and abdomen on the participants’ dominant hand side (Figure 1(c)). Muscle activity was measured with surface electromyography (EMG) using Delsys sensors (Delsys Trigno, Delsys Europe, United Kingdom). Sensors were placed according to SENIAM guidelines (Stegeman and Hermens, 2007). Activity of the following movement agonists was measured: Biceps Brachii (BB), Deltoideus_anterior_ (AD), and Deltoideus_medius_ (MD). Activity of the following antagonist and related muscles was measured: Triceps Brachii (TB), Deltoideus_posterior_ (PD), Trapezius Descendens (TR), Latissimus Dorsi (LD), Erector Spinae_lumbar_ (ES), and Rectus Abdominis (RA).

To obtain MVC normalization values the following exercises were performed, with the targeted muscle(s) indicated in brackets: Scapular elevation in sitting posture (TR) (Boettcher et al., 2008), 90 shoulder flexion (AD) (Kim et al., 2018), 90° shoulder abduction (MD, PD) (Boettcher et al., 2008), prone spinal extension (ES) (Al-Qaisi et al., 2021), prone arm extension (LD) (Park and Yoo, 2013), seated elbow flexion (BB) (Stegeman and Hermens, 2007), seated elbow extension (TB) (Stegeman and Hermens, 2007) and isometric curl-up (RA) (Lehman and McGill, 2001). During the exercise targeting the Erector Spinae, gravity provided resistance to the movement, and the participants were restricted in their range of motion in a prone posture. During all other exercises, resisting force was applied by the experimenter. The MVC attempts lasted 10 s, during which participants received verbal encouragement.

Data visualization and statistical testing were performed using Matlab 2019b (MathWorks, USA). The EMG signals were filtered according to SENIAM guidelines using a bidirectional 4th order butterworth bandpass filter (Fc: 10 Hz and 500 Hz). Multiple infinite impulse response notch filters (50, 150, 222, 296, 370, and 444 Hz) with *Q* factor 20 were applied to remove noise at these frequencies observed in the spectral plots. The root mean square (RMS) muscle activity during the entire duration of a task is reported as outcome measures.

#### 2.4.2. Cardiac cost

Heart rate was measured continuosly throughout the experiment using an optical wristwatch (Polar Ignite 2, Polar Electro Europe AG, Germany). The resting heart rate was calculated as the mean heart rate of a 2 min period where the participant rested on a chair. An exponential curve was fitted through the heart rate data for each individual, for each task and condition. The cardiac cost was calculated as the value of the exponential curve at the end of the task normalized to the resting heart rate.

#### 2.4.3. Subjective data

After each quasi-isometric and dynamic task, the participant rated their perceived exertion and task-related discomfort. The latter was assessed for the following six body parts: neck, shoulder, chest, upper back, upper arm, and lower back. Perceived constraint was queried if the exoskeleton was used for the task. The questionnaires used a CR-10 Borg scale (Borg, 1990). For all questions, 0 was the lowest answer and referred to no exertion, task-related discomfort, or constraints, and 10 was the highest answer and referred to maximal exertion, task-related discomfort, or constraint.

After completing all tasks, participants rated exoskeleton usability using the validated System Usability Scale, which includes 10 questions rated on a 5-point Likert scale (Bangor et al., 2008). The possible answers ranged from 1 (strongly disagree) to 5 (strongly agree).

### 2.5. Statistics

Repeated measures ANOVA and paired sample t-tests were used to compare the exoskeleton conditions for the continuous data (muscle activity and cardiac cost). Normality of the sampling distribution of the mean was assumed based on the sample size (n = 32) being large enough according to central limit theorem. Effects of the repeated measures ANOVA and the paired sample t-tests were considered significant if the *p*-value was <0.05. If the repeated measures ANOVA was significant, post-hoc analysis was conducted using paired sample *t*-tests comparing OFF-SL1, OFF-SL2, and SL1-SL2 conditions. For post-hoc analysis the significance level was Bonferonni corrected for the three comparisons and effects were considered significant when the *p*-value was <0.0165. Wilcoxon signed-rank tests were used for the discrete questionnaire data. Effects of the Wilcoxon signed-rank test were considered significant if the *p*-value was <0.05.

## 3. Results

### 3.1. Effect of support level on muscle activity

**Figure 3.**
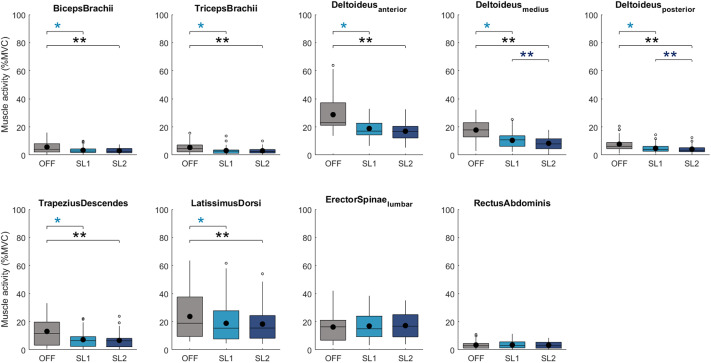
Change in muscle activity RMS amplitude as a percent of maximal voluntary contraction (%MVC) between the no exoskeleton (OFF) in grey, exoskeleton support level 1 (SL1) in light blue, and support level 2 (SL2) condition in blue in the isometric task. The data are displayed as box plots, with a dot representing the mean value. Paired *t*-test *p*-values are reported. *p < 0.0165 and **p < 0.0033.

During the isometric task with 90° shoulder flexion and extended elbow, the factor exoskeleton condition significantly influenced muscle activity of the Biceps Brachii, the Triceps Brachii, all three heads of the Deltoideus (anterior, medius, posterior), the Trapezius Descendens, and the Latissimus Dorsi (*p*_ANOVA_ *<* 0.001). For these muscles, exoskeleton use resulted in significant reductions in activity regardless of the chosen support level (Figure 3). Moreover, the SL2 provided by the exoskeleton resulted in a further significant reduction in muscle activity for all three heads of the Deltoideus (anterior: *p*_SL1-SL2_ *<* 0.0165, medius: *p*_SL1-SL2_ *<* 0.0033, posterior: *p*_SL1-SL2_ *<* 0.0033) compared to the SL1.

The Deltoideus_medius_ muscle activity was reduced from 16.7% MVC while performing the task without exoskeleton support to 9.8% MVC with SL1 ( 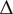 RMS: 41%, p_OFF-SL1_ *<* 0.0033) and further decreased to 7.8% MVC with SL2. Switching from SL1 to SL2 resulted in an additional RMS reduction of 20% (p_SL1-SL2_ *<* 0.0033) for the Deltoideus_medius_. When comparing the different conditions of the performed isometric task, there was no significant change in muscle activity for the Erector Spinae and the Rectus Abdominis.

The other four isometric postures performed in the study yielded similar results (Supplementary Material Table 1). Overall, when comparing working with the exoskeleton at SL2 to the no exoskeleton condition, the highest reduction of muscle activity in the Trapezius Descendens was found for the 120° shoulder flexion with extended elbow posture by 56% (p_OFF-SL2_ *<* 0.0033), and in the Deltoideus_medius_ for the 90° shoulder and elbow flexion posture by 64% (p_OFF-SL2_ *<* 0.0033).

### 3.2. Muscle activity during occupational tasks

**Figure 4.**
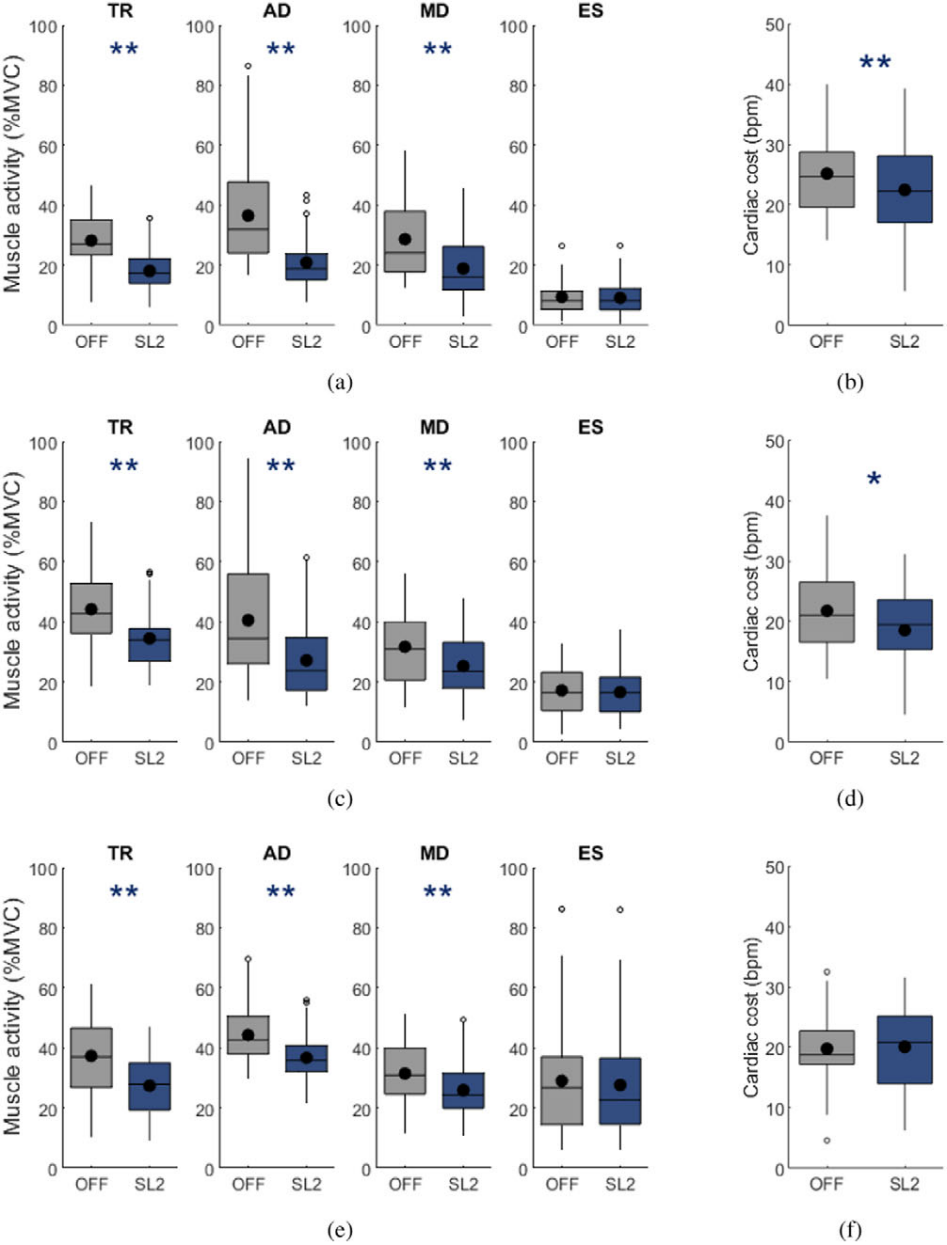
Change in muscle activity RMS amplitude as a percent of maximal voluntary contraction (%MVC) between the no exoskeleton (OFF) in grey and support level 2 (SL2) condition in blue during (a) assembly, (c) power tool handling and (e) load manipulation tasks for the four muscles that benefit most from the exoskeleton support: Trapezius Descendens (TR), Deltoideus_anterior_ (AD), Deltoideus_medius_ (MD) and Erector Spinae_lumbar_ (ES). Change in cardiac cost between the no exoskeleton (OFF) in grey and exoskeleton support level 2 (SL2) condition in blue in the (b) assembly, (d) power tool task, and (f) load manipulation tasks. The data are displayed as box plots, with a dot representing the mean value. Paired *t*-test *p*-values are reported. *p < 0.05 and **p < 0.01.

In all three quasi-isometric and dynamic tasks, significant reductions of muscle activity in the Biceps Brachii (*p <* 0.01), the Trapezius Descendens (*p <* 0.001), and all three heads of the Deltoideus (anterior: *p <* 0.001, medius: *p <* 0.001, posterior: *p <* 0.001) were observed when working with the exoskeleton compared to working without the exoskeleton (Figure 4). Further, the RMS muscle activity in the Erector Spinae and Rectus Abdominis did not differ between conditions in any tasks (Table 1).

**Table 1.** Table with results of the quasi-isometric and dynamic tasks. Reported are the mean (M) and standard deviations (SD) of the RMS muscle activity during the no exoskeleton (OFF) and support level 2 (SL2) conditions, the change in RMS in (%OFF) calculated as (M_OFF_ – M_SL2_)/M_OFF_*100, the number of participants included in each analysis (n) and the p-value of the paired t-test.

	RMS 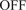 (%MVC)		RMS 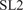 (%MVC)	
	M	SD	M	SD	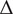 RMS (%OFF)	n	*p*-value
***Assembly***							
Biceps Brachii	7.6	7.0	4.6	3.9	40.1	32	<0.001
Triceps Brachii	5.5	3.4	4.7	2.8	14.3	29	<0.01
Deltoideus_anterior_	36.6	17.2	21.0	9.1	42.7	31	<0.001
Deltoideus_medius_	28.7	12.7	18.9	10.2	34.1	31	<0.001
Deltoideus_posterior_	18.1	11.4	15.2	10.6	15.8	30	<0.05
Trapezius Descendens	28.2	8.9	18.1	6.3	35.8	31	<0.001
Erector Spinae_lumbar_	9.4	5.9	9.2	5.8	3.0	26	0.776
Latissimus Dorsi	25.6	18.4	23.8	16.7	6.8	29	0.132
Rectus Abdominis	4.1	2.7	3.8	2.5	6.4	29	0.059
***Power tool handling***							
Biceps Brachii	12.0	9.3	9.9	7.0	17.5	28	<0.01
Triceps Brachii	13.2	6.8	14.4	8.6	−8.8	26	0.307
Deltoideus_anterior_	40.5	19.3	27.2	12.3	32.9	28	<0.001
Deltoideus_medius_	31.7	13.0	25.2	10.0	20.3	29	<0.001
Deltoideus_posterior_	29.8	16.6	26.6	13.7	10.8	27	<0.05
Trapezius Descendens	44.2	13.6	34.5	8.7	21.9	29	<0.001
Erector Spinae_lumbar_	17.2	8.2	16.6	8.3	3.6	22	0.627
Latissimus Dorsi	33.0	20.4	32.7	20.2	1.0	30	0.854
Rectus Abdominis	6.6	4.1	7.1	3.9	−7.3	29	0.456
***Load manipulation***							
Biceps Brachii	20.4	8.8	18.1	7.8	11.4	32	<0.001
Triceps Brachii	16.9	11.9	14.4	8.5	15.1	24	<0.05
Deltoideus_anterior_	44.2	8.9	36.7	8.4	17.0	26	<0.001
Deltoideus_medius_	31.5	10.7	25.9	9.6	17.6	29	<0.001
Deltoideus_posterior_	23.4	15.1	19.8	13.0	15.3	28	<0.01
Trapezius Descendens	37.3	11.8	27.4	10.0	26.6	31	<0.001
Erector Spinae_lumbar_	29.1	19.8	27.6	18.7	4.9	28	0.167
Latissimus Dorsi	34.4	19.6	33.6	19.7	2.3	27	0.626
Rectus Abdominis	5.0	3.3	4.8	3.5	3.6	27	0.691

In the assembly task the main muscles that benefited from exoskeleton support were the Deltoideus_anterior_ ( 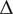 RMS: 42.7%, *p <* 0.001), the Biceps Brachii ( 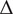 RMS: 40.1%, *p <* 0.001), the Trapezius Descendens ( 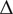 RMS: 35.8%, *p <* 0.001) and the Deltoideus_medius_ ( 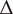 RMS: 34.1%, *p <* 0.001), see Figure 4(a). Additionally, significant reductions in Triceps Brachii and Deltoideus_medius_ activity were observed, while the muscles in the lower back and abdomen were not influenced by exoskeleton use (Table 1).

The power tool handling task required more muscle activity than the assembly task, as is evident by the higher RMS values in the no exoskeleton conditions of the assembly and tool handling tasks. The exoskeleton support significantly reduced the muscle load in the Trapezius Descendens and the three Deltoideus. The neck muscle activity was reduced by 21.9% (*p <* 0.001) in the SL2 condition from executing the task without the exoskeleton. The reduction in Deltoideus activity ranged from 10.8% (*p <* 0.05) in the posterior head to 32.9% (*p <* 0.001) in the anterior head (Figure 4(c) and Table 1).

The Trapezius Descendens activity was reduced by 26.6% (*p <* 0.001) when working with the DeltaSuit in the load manipulation task. The reduction in the shoulder muscles ranged from the Deltoideus_posterior_ with 15.3% (*p <* 0.01) up to 17.6% (*p <* 0.001) in the Deltoideus_anterior_ (Figure 4(e) and Table 1).

### 3.3. Cardiac cost

The support provided by the exoskeleton significantly reduced the cardiac cost for the assembly (*p <* 0.001) and power tool handling (*p <* 0.05) tasks, but not for the load manipulation task (n.s., *p =* 0.746), see Figure 4(f). During the assembly task, the cardiac cost was reduced from 25.2 bpm (SD: 6.7 bpm) without support to 22.5 bpm (SD: 7.7 bpm) in the SL2 condition, resulting in a reduction of 11% (Figure 4(b)). When using a power tool a reduction of 3.2 bpm (OFF: 21.9 bpm (SD: 6.8 bpm), SL2: 18.7 bpm (SD: 7.6 bpm)) could be seen, amounting to a reduction in cardiac cost of 15%.

### 3.4. Subjective data

The perceived exertion was reduced in all quasi-isometric and dynamic tasks (Table 2). The mean reduction over all tasks was 21.5%OFF (*p <* 0.001). The task-related discomfort was reduced in the shoulder (57%OFF, *p <* 0.001), the upper arm (25%OFF, *p <* 0.05), and the lower back (76%OFF, *p <* 0.05). The constraint was rated overall tasks as 2.4/10, which corresponds to little constraint.

**Table 2.** Mean of reported perceived-exertion (RPE), task-related discomfort (RPD), and constraint (RPC) for the no exoskeleton (OFF) and support level 2 (SL2) condition in the quasi-isometric and dynamic tasks, the change ( 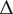 (%OFF)) in the subjective ratings overall task calculated as (M_OFF_ – M_SL2_)/M_OFF_*100 and the p-values of the Wilcoxon signed-rank test of the mean values of the conditions overall tasks.

	Assembly		Power tool handling		Load manipulation		M_OFF_ – M_SL2_ all tasks	
	M 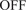	M 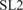	M 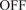	M 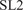	M 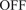	M 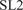	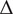 (%OFF)	*p*-value
Exertion	5.1	3.8	6.9	5.6	4.2	3.3	21.5	< 0.001
Task-related discomfort
Neck	0.7	0.3	0.7	0.4	0.2	0	59	0.168
Shoulder	3.0	1.1	2.9	1.1	1.7	1.1	57	<0.001
Chest	0	0	0	0	0.1	0	74	0.500
Upper Back	0.2	0.2	0.6	0.3	0.4	0.5	26	0.576
Upper arm	2.1	1.6	3.2	1.7	0.8	1.2	25	<0.05
Lower back	0.2	0	0.2	0.2	0.9	0.1	76	<0.05
Constraint	N.A.	2.4	N.A.	2.3	N.A.	2.6	N.A.	N.A.

### 3.5. Usability

The mean system usability score was 87 (IQ1: 80, IQ3: 95, SD: 8.6). The highest possible score is 100, whereas scores above 85.58 are considered an excellent rating (Bangor et al., 2008). On the questions regarding system complexity, ease of use, need for technical assistance, ease of learning to use this system, and the need to learn a lot of things before getting going with the system, the mean of the answers had the highest possible score.

### 3.6. Biometric characteristics and exoskeleton fit

**Figure 5.**
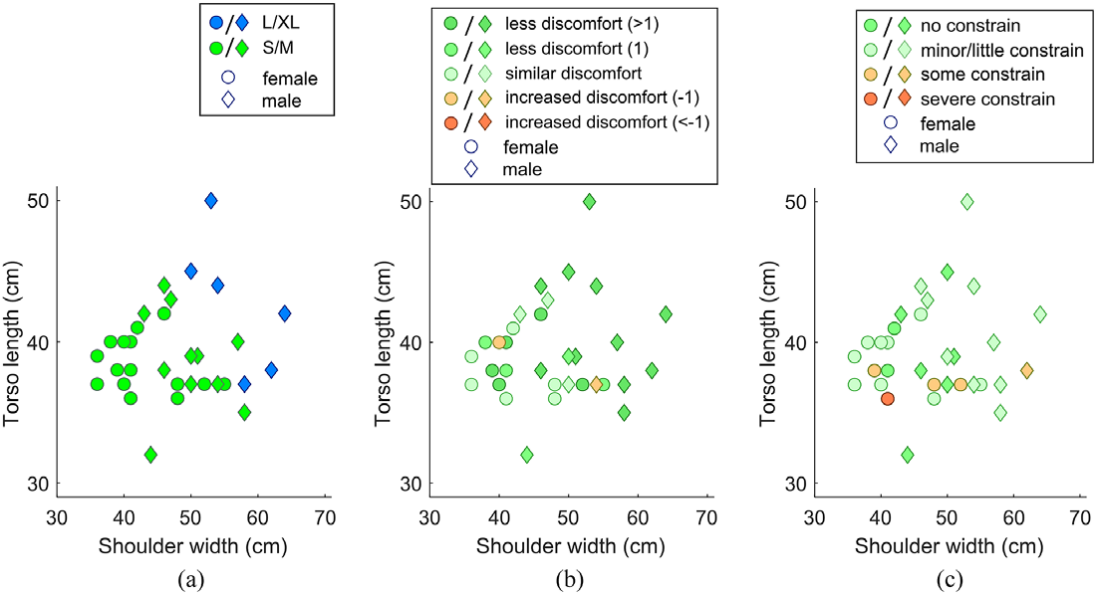
Relationship between participant shoulder width, torso length, gender and (a) distribution of exoskeleton size (S/M or L/XL), (b) support-related change in task-related discomfort in the shoulder or (c) perceived constrain.

Shoulder width and torso length were the main biometric characteristics that determined exoskeleton size choice. Six participants (all male) were fitted in the L/XL size, 26 participants (11 male, 15 female) were fitted with the S/M size (Figure 5(a)). Shortening the exoskeleton back structures would likely change the distribution of the fit to a more equal split between S/M and L/XL. The majority of participants experienced less task-related discomfort in the shoulder when performing the quasi-dynamic and dynamic tasks with exoskeleton support (21 of 32), while nine participants did not experience task-related discomfort in the shoulder during the OFF condition or did not benefit from shoulder support. Two participants (one male, one female) reported a slight increase in task-related discomfort in the shoulder (Figure 5(c)). A potential mismatch between torso length, shoulder width, and exoskeleton size did not seem to explain perceived constraint (Figure 5(c)).

## 4. Discussion

We set out to evaluate a novel passive exoskeleton providing shoulder support using an evaluation protocol designed according to benchmarking recommendations considering various tasks selected for their relevance in work conditions, as well as quantitative and qualitative outcome measures. The DeltaSuit is designed to be very lightweight allowing high freedom of movement while providing relevant support during overhead tasks. As reported for other upper limb occupational exoskeletons we hypothesized that working with the novel passive exoskeleton would reduce muscle activity and task-related discomfort, while inducing minimal movement constrain. Due to the low weight of the system we hypothesized the reductions in muscle activity would result in reduction in cardiac cost and perceived exertion.

The activation of shoulder flexor muscles was significantly reduced during multiple overhead tasks, which was accompanied by reductions in antagonist muscles, implying reduced co-contraction. More specifically, during the isometric task, a decrease in muscle activity of up to 56% was observed in the Trapezius Descendens and up to 64% in the Deltoideus_medius_ when working with the exoskeleton. In the quasi-isometric and dynamic tasks, the exoskeleton provided relevant support with a reduction in neck muscle activity of up to 34% and a decrease in Deltoideus muscle activity of up to 36%. These reductions in muscle activity were associated with reduced task-related shoulder discomfort (57%), cardiac cost (up to 15%), and perceived exertion (22%). This confirms our previous work reporting a reduction in muscle fatigue of up to 45% (*p <* 0.01) in the Deltoideus_anterior_ when using the DeltaSuit for overhead work (Brunner et al., 2023).

Direct comparisons of our findings to literature evaluating passive overhead systems were challenging due to the wide variety of study protocols and reported outcome measures in other studies. It is important to consider the variation in the task design, recording settings, normalization protocols, and choice of outcome measures when comparing performance of different systems. We chose to create our evaluation protocol in line with recommendations by De Bock et al. (2022), as the suggestions in this work are based on an extensive analysis of the literature on occupational exoskeletons to date and the proposed protocols for laboratory evaluation resemble occupational tasks observed in the field. One remark to the use of the proposed standardized overhead height is that this position does not correspond to the position with peak gravitational torque around the shoulder, and thus for most exoskeleton designs is not the position where the user receives maximum support. For the isometric task, we therefore included both 90° shoulder flexion and standardized overhead height positions.

The changes in agonist muscles involved in overhead movements during the isometric and quasi-isometric tasks are in the similar range to those reported in previous studies evaluating passive shoulder exoskeleton support. Muscle activity recordings during the isometric task align with those of Pacifico et al., 2020, who maintained a 90° flexion with an extended elbow posture for 60 s without any additional load, similar to our study. The Proto-MATE reduces the Deltoideus up to 18% (Pacifico et al., 2020) whereas in our study a reduction of 43 to 64% could be seen. It is likely that the lighter device weight of the DeltaSuit and higher support explain the difference in observed Deltoideus reductions. Our assembly task protocol is comparable to other overhead tasks without external load (Kim et al., 2018; Kim and Nussbaum, 2019; Grazi et al., 2020; Schmalz et al., 2019). Reported Deltoideus_anterior_ reductions range from 38% in the Mate Prototype (Grazi et al., 2020) to 53% in the EksoVest (Kim et al., 2018). With 43% Deltoideus_anterior_ reduction the shoulder support provided by the DeltaSuit falls within the reported range. Numerous studies have investigated exoskeletons in the context of an overhead task with a power tool (Hyun et al., 2019; Yin et al., 2019; Schmalz et al., 2019; Maurice et al., 2019; De Bock et al., 2023; Kim et al., 2018; Kim and Nussbaum, 2019). The tool weight ranged from 0.66 kg (Maurice et al., 2019) to 5.9 kg (Kim et al., 2018) with an average weight of 2.3 kg. The muscle load reduction in the Deltoideus_anterior_ ranged from 16% (Kim et al., 2018) in the EksoVest up to 52% in the PAEXO (Maurice et al., 2019). With the DeltaSuit, a reduction of 33% could be shown.

Changes in agonist muscles involved in overhead movements during dynamic tasks can be compared to Theurel et al. (2018) and van der Have et al. (2022). Deltoideus_anterior_ varied greatly between exos and studies, from 2% with the Exo4Work (van der Have et al., 2022) to 54% with the Exhauss Stronger (Theurel et al., 2018). With 17% Deltoideus_anterior_ reduction the DeltaSuit is mid-range. In addition, the DeltaSuit reduced Trapezius Descendes activity by 27%, which was very similar to the reduction reported for the Exo4Work when lifting loads over shoulder level (van der Have et al., 2022). The Exhauss Stronger, Exo4Work, and DeltaSuit vary significantly in size and weight, with the Exhauss being the largest and heaviest system (9 kg) and the DeltaSuit being the lightest (2.1 kg). It is important to consider the trade-off between size, weight, and support in exoskeletons. Although heavier and larger exoskeletons can potentially provide more support, they are likely to impose more constraints, ultimately affecting performance, comfort and user experience. Conversely, smaller exoskeletons such as the DeltaSuit demonstrate fewer constraints. A variety in exoskeleton designs allows to accommodate for the diverse use cases encounter in different industries (De Vries and De Looze, 2019).

We further observed significant activity reductions in antagonist muscles including the Triceps Brachii and the Deltoideus_posterior_. Besides providing support, the rigid structures in the exoskeleton have a stabilizing function reducing the need for co-contraction. Activity reductions in antagonist muscles are especially important because it shows the user is not fighting the exoskeleton support and having less co-contraction may reduce load in the respective joints. For example, increased Triceps Brachii was shown during load manipulation with the Exhauss Stronger (Theurel et al., 2018), implying participants of that study might have had to work against the supportive force of the exoskeleton. Deltoideus activity influences the compression forces acting in the glenohumeral joint, which during dynamic overhead work moves along a combined flexion/abduction and extension/adduction trajectory. During this type of movement the activation of the Deltoideus leads to a vertical dislocation force in the glenohumeral joint, which is naturally stabilized by simultaneous activation of the rotator cuff muscles. When working with a shoulder exoskeleton, the Deltoideus muscle activity can be reduced and consequently the glenohumeral joint compression forces decreased, which may impact the prevalence and also the treatment of existing shoulder musculoskeletal disorders (Schmalz et al., 2019).

To assess possible adverse effects we also focused on the Erector Spinae and Rectus Abdominis activity, which in this study were not affected by exoskeleton use in any of the tasks. The literature reveals a broad range of effects on the Erector Spinae when using a shoulder exoskeleton. For example an increase of 50% is reported by Hyun et al. (2019) whereas Kim and Nussbaum (2019) showed a 25% reduction during load manipulation tasks. With the DeltaSuit, we observed a modest reduction in lower back activity during load manipulation, confirming that the design choice of attaching the exoskeleton to the upper torso instead of the hip, did not negatively impact loading on the lower back.

When muscles use less oxygen, their demand on the cardiovascular system goes down, explaining why the observed muscle activity reductions in this study were accompanied by reductions in cardiac cost. For overhead work without external load, Schmalz and colleagues reported a reduction of 21% in cardiac cost (6 bpm; Schmalz et al., 2019), whereas Grazi et al. report a reduction of 8% in absolute heart rate (Grazi et al., 2020). In comparison, we observed a reduction of 11% in cardiac cost (2.7 bpm) when using the DeltaSuit to support work with no external load, which falls in the range of these previously reported results. When handling a power tool, our study reveals a reduction of 15% in cardiac cost (3.2 bpm), which is smaller than the 28% of cardiac cost (7 bpm) reduction reported by Schmalz et al. (2019). Participants in their study performed 10 min of task execution, which gives the heart rate more time to stabilize (compared to 3 min in our study), resulting in a very accurate cardiac cost estimate. Lastly, the cardiac demand associated with the dynamic load manipulation task can be compared to Theurel et al. (2018), who reported a 14% increase in cardiac cost (7.2 bpm), whereas no significant effect of exoskeleton support was found in this study. It is probable that besides differences in exoskeleton support level and study protocol, the weight of the evaluated systems contributes to differences in cardiac demand.

The reported physiological benefits of exoskeleton use seem perceivable to the participants, as is evident by a 21.5% decrease in perceived exertion and a 53% reduction in task-related discomfort across all body parts. The overall constraint when working with the DeltaSuit was minimal, with 27 of 32 participants reporting little to no constraints. The results obtained from the rating of perceived exertion can be compared to findings of Desbrosses et al. (2021), who reported a reduction of 3.2/10 in upper limb exertion when using the Exhauss or Skelex exoskeleton, and to findings of Grazi et al. (2020), who reported an exertion reduction of 3/10 when using the H-Pulse. The task-related discomfort in the shoulder can be compared to findings of Alabdulkarim and Nussbaum (2019), who reported a similar rating for no exoskeleton and exoskeleton (FORTIS, SuitX and Fawcett Exovest) conditions. Further, Kim and Nussbaum (2019) reported a reduction in shoulder discomfort from moderate (3/10) to mild (2/10) discomfort when maintaining overhead height, which is similar to the decrease in shoulder discomfort from moderate (2.5/10) to minor (1.1/10) reported in the current study.

Participants with a range of body types and sizes (height: 160 - 194 cm; weight: 43.8 - 98.1 kg) could be fit into the exoskeleton and provided with physiologically meaningful support in a comfortable manner. In this sample, only males were fitted into the L/XL size. Based on these findings the final product vest and back-structure length were shortened to improve fit and size distribution. Even though, the device DoF do not replicate those of the human shoulder joint, the average reported constraint was little to minor. Specifically, decoupling the left and right rigid structures and not restricting spinal rotation and flexion/extension, allows the user to access their torso range of motion while wearing the exoskeleton. Experimenters observed occasional displacement of the vertical rotation joint of the shoulder mechanism, which seemed to be related to the complaints of constraint in a subgroup of participants (5 of 32 participants) during the dynamic task. This could be solved after the study, through additional stabilization of the vertical rotation axis of the shoulder mechanism.

When switching DeltaSuit support from SL1 (5.2 Nm) to SL2 (6.6 Nm) the user received 20% more supportive torque around the shoulder joint. As we observed a corresponding decrease in medial Deltoideus muscle activity of 20%, it could be concluded that this additional supportive torque is effectively transmitted to the user.

While our findings confirm the physiological and experienced benefits of using this novel shoulder exoskeleton, there are some limitations in the experimental design, which emphasize the need to further evaluate the exoskeleton. The heart rate monitor used in this study is not a research-grade physiological monitoring device. While it has been validated as a heart rate measurement device during activities of moderate exercise intensity (Budig et al., 2021), the reported heart rate values might underestimate the heart rate of our participants (Jagim et al., 2020). Nonetheless, the comparison between baseline and exoskeleton heart rate is unlikely to be influenced by this discrepancy. Further, the study was conducted in the laboratory under controlled conditions and the participants were unfamiliar with exoskeleton use and repeated overhead work. Additionally, it would be recommended to provide longer periods for familiarization in future studies, as routinized human movements are typically efficient, and wearing an exoskeleton might initially disturb this (Moeller et al., 2022). With proper familiarization and prolonged use, the user’s movements are likely to adapt to the support provided by the exoskeleton (Moeller et al., 2022). Further investigations should be conducted in the field with target end-users to see how the performance indicators obtained using the evaluation protocol transfer to occupational settings.

## 5. Conclusion

The DeltaSuit exoskeleton has been found to effectively alleviate the muscle load on the arm, neck and shoulder muscles during various overhead tasks without causing an increase in load on other muscles. Additionally, working with the DeltaSuit has been associated with lowered cardiac cost, reduced user exertion, and decreased task-related discomfort while imposing minimal constraints on users. Taken together, these results confirm that passive exoskeletons such as the DeltaSuit have the potential to significantly support users when performing tasks in overhead postures and are a good solution to relieve the critical body parts of biomechanical strains for workers at high risk of musculoskeletal disorders.

## Supporting information

Brunner et al. supplementary material Brunner et al. supplementary material

## Supplementary material

The supplementary material for this article can be found at http://doi.org/10.1017/wtc.2023.21.
