# Supplementary material for: Effect of passive shoulder exoskeleton support during working with arms over shoulder level
Source: Wearable Technol. 2023 Nov 3;4:e26. doi: 10.1017/wtc.2023.21 (PMC10952051; doi:10.1017/wtc.2023.21)
Supplement: Brunner et al. supplementary material [file S263171762300021Xsup001.pdf]

6. Supplementary material

Supplementary Table 1. Table with results of the isometric task. Reported are the mean (M) and standard deviations (SD) of the RMS muscle activity during the no exoskeleton (OFF), support level 1 (SL1) and support level 2 (SL2) conditions, the number of participants included in each analysis (n) and the p-value of the repeated measures ANOVA.

	RMS_{OFF} (%MVC)		RMS_{SL1} (%MVC)		RMS_{SL2} (%MVC)				
	M	SD	M	SD	M	SD	ΔRMS_{SL2} (%OFF)	n	p-value
90° shoulder and elbow flexion									
Biceps Brachii	4.8	4.3	2.0	1.8	1.9	1.4	61	29	<0.001
Triceps Brachii	3.6	2.4	2.7	1.9	2.8	2.2	23	29	<0.005
$Deltoideus_{anterior}$	27.9	9.0	17.6	9.1	15.3	9.5	45	29	<0.001
$Deltoideus_{medius}$	16.7	8.5	8.5	6.6	6.0	4.8	64	31	<0.001
$Deltoideus_{posterior}$	6.2	4.0	3.8	2.5	3.6	3.1	43	30	<0.001
Trapezius Descendens	8.4	7.4	5.0	5.0	4.2	4.7	50	27	<0.001
Erector $Spinae_{lumbar}$	12.9	7.4	12.7	8.6	12.8	7.1	1	24	0.978
Latissimus Dorsi	24.1	18.9	17.5	11.6	17.0	11.3	29	30	<0.001
Rectus Abdominis	3.5	3.0	3.4	2.9	3.2	2.4	8	30	0.587
120° shoulder flexion									
Biceps Brachii	4.9	3.9	2.7	2.2	2.4	1.8	51	29	<0.001
Triceps Brachii	4.2	3.2	3.0	2.2	3.4	2.8	20	28	<0.005
$Deltoideus_{anterior}$	30.2	10.7	18.7	10.2	16.7	9.1	45	29	<0.001
$Deltoideus_{medius}$	21.2	8.7	12.8	9.0	10.4	8.2	51	32	<0.001
$Deltoideus_{posterior}$	8.9	4.4	6.1	3.9	5.7	4.1	36	30	<0.001
Trapezius Descendens	16.0	9.4	8.6	6.5	7.1	6.1	56	30	<0.001
Erector $Spinae_{lumbar}$	12.0	7.2	12.0	6.7	12.0	6.6	0	21	0.999
Latissimus Dorsi	24.5	19.8	18.7	13.2	17.7	11.8	28	30	<0.001
Rectus Abdominis	3.4	2.7	3.2	2.6	3.2	2.3	6	30	0.522
90° shoulder and elbow flexion, abduction									
Biceps Brachii	8.0	8.8	5.2	5.4	4.5	4.2	44	24	<0.001
Triceps Brachii	4.9	4.7	3.4	2.7	3.0	2.3	40	30	<0.001
$Deltoideus_{anterior}$	31.6	16.7	20.7	12.9	18.5	11.9	42	30	<0.001
$Deltoideus_{medius}$	21.4	14.8	14.8	14.0	11.0	11.4	49	32	<0.001
$Deltoideus_{posterior}$	10.8	9.0	7.4	7.6	5.6	5.5	48	31	<0.001
Trapezius Descendens	16.3	7.0	9.4	5.5	7.9	5.0	51	27	<0.001
Erector $Spinae_{lumbar}$	11.3	7.2	11.0	7.1	10.9	5.8	4	22	0.942
Latissimus Dorsi	23.4	15.7	18.5	11.7	17.5	11.1	25	31	<0.001
Rectus Abdominis	3.5	2.8	3.3	2.6	3.3	2.4	6	30	0.674
120° shoulder, 90° elbow flexion, abduction									
Biceps Brachii	5.1	5.2	3.7	3.5	3.5	3.2	32	27	<0.001
Triceps Brachii	4.2	3.2	3.4	2.3	3.1	2.0	24	31	<0.05
$Deltoideus_{anterior}$	34.1	13.8	25.5	11.8	22.7	10.4	34	28	<0.001
$Deltoideus_{medius}$	21.6	7.9	17.3	10.3	14.2	9.0	34	28	<0.001
$Deltoideus_{posterior}$	10.8	6.7	8.3	6.0	7.4	6.0	32	30	<0.001
Trapezius Descendens	19.5	8.4	11.7	7.0	9.9	5.7	49	28	<0.001
Erector $Spinae_{lumbar}$	11.8	8.3	11.2	7.2	11.7	7.3	1	24	0.807
Latissimus Dorsi	25.4	18.9	21.5	14.9	20.7	14.6	19	30	<0.005
Rectus Abdominis	3.5	2.7	3.4	2.6	3.3	2.5	6	30	0.598
